# Systems analysis of transcription factor activities in environments with stable and dynamic oxygen concentrations

**DOI:** 10.1098/rsob.120091

**Published:** 2012-07

**Authors:** Matthew D. Rolfe, Andrea Ocone, Melanie R. Stapleton, Simon Hall, Eleanor W. Trotter, Robert K. Poole, Guido Sanguinetti, Jeffrey Green

**Affiliations:** 1 Department of Molecular Biology and Biotechnology, The Krebs Institute, University of Sheffield, Sheffield S10 2TN, UK; 2 School of Informatics, University of Edinburgh, Edinburgh ED8 9AB, UK; 3 SynthSys—Synthetic and Systems Biology, University of Edinburgh, Mayfield Road, Edinburgh EH9 3JD, UK

**Keywords:** *Escherichia coli*, mathematical modelling, oxygen-sensing, systems biology, transcript profiling

## Abstract

Understanding gene regulation requires knowledge of changes in transcription factor (TF) activities. Simultaneous direct measurement of numerous TF activities is currently impossible. Nevertheless, statistical approaches to infer TF activities have yielded non-trivial and verifiable predictions for individual TFs. Here, global statistical modelling identifies changes in TF activities from transcript profiles of *Escherichia coli* growing in stable (fixed oxygen availabilities) and dynamic (changing oxygen availability) environments. A core oxygen-responsive TF network, supplemented by additional TFs acting under specific conditions, was identified. The activities of the cytoplasmic oxygen-responsive TF, FNR, and the membrane-bound terminal oxidases implied that, even on the scale of the bacterial cell, spatial effects significantly influence oxygen-sensing. Several transcripts exhibited asymmetrical patterns of abundance in aerobic to anaerobic and anaerobic to aerobic transitions. One of these transcripts, *ndh*, encodes a major component of the aerobic respiratory chain and is regulated by oxygen-responsive TFs ArcA and FNR. Kinetic modelling indicated that ArcA and FNR behaviour could not explain the *ndh* transcript profile, leading to the identification of another TF, PdhR, as the source of the asymmetry. Thus, this approach illustrates how systematic examination of regulatory responses in stable and dynamic environments yields new mechanistic insights into adaptive processes.

## 2. Introduction

The bacterium *Escherichia coli* K-12 is a key model organism that is able to grow in the presence and absence of oxygen. It is biochemically versatile, having three basic metabolic modes—aerobic respiration, anaerobic respiration and fermentation [1–3]. Each metabolic mode has the potential to conserve different amounts of energy, and hence the most efficient, aerobic respiration, is preferred to anaerobic respiration, which is in turn preferred to the least efficient metabolic mode, fermentation. Under carbon-limited conditions, oxygen availability is the major determinant of which metabolic mode is adopted [1] and, as is evident from the profound changes in biochemistry noted earlier, the response to changes in oxygen availability requires significant reprogramming of *E. coli* K-12 gene expression [4–6].

*Escherichia coli* K-12 has two major oxygen-sensing transcription factors (TFs): the indirect oxygen sensor ArcBA and the direct oxygen sensor FNR [7]. Together, these regulators optimize growth in the presence or absence of oxygen by remodelling central metabolism. Consequently, regulatory circuits that react to key metabolic signals must be integrated into the bacterial response to oxygen. Moreover, oxygen availability alters the properties of some nutrients (e.g. the redox state of metal ions), which in turn acts as a signal to other regulatory circuits. To fully understand the complex regulatory remodelling underpinning responses to changes in oxygen availability requires detailed knowledge of the changes in activity of multiple TFs. Experimental measurement of the activities of numerous TFs in a dynamic environment is unfeasible, however, owing to technical limitations. Therefore, statistical approaches have been proposed to infer changes in TF activities from downstream target data [8–11]. While these models rely on simplifying assumptions, they have been shown to yield non-trivial and verifiable predictions for individual TFs [4,12,13]. Here, transcript profiling, mathematical modelling and model validation have been used to systematically study *E. coli* K-12 TF activities in stable (steady-state) environments maintained at fixed oxygen supply rates, and in the unstable dynamic environments created during transitions between aerobic and anaerobic conditions.

## 3. Material and methods

### 3.1. Strains and chemostat growth conditions

*Escherichia coli* K-12 MG1655 and its derivatives JRG6009 (a *lac* mutant carrying the FF(-41.5) FNR-reporter plasmid [14]) and JRG6031 (*pdhR* mutant) were used.

Steady-state continuous cultures were established in a 2 l Labfors chemostat (Infors-HT, Bottmingen, Switzerland) in glucose-limited Evans Medium [4,15]. Steady-state chemostat cultures at different aerobiosis levels were established as described previously [4,16]. Anaerobic conditions were sustained by sparging with 5% CO_2_/95% N_2_. Transitions were carried out by adjusting the gas supply. Dissolved oxygen levels were monitored using a TruDO Dissolved Oxygen Sensor (Finesse). β-Galactosidase assays were carried out according to Miller [17].

### 3.2. RNA isolation

Chemostat culture samples for transcriptional profiling were directly eluted into RNAprotect (Qiagen) to rapidly stabilize the mRNA. Total RNA was prepared using the RNeasy RNA purification kit (Qiagen), according to the manufacturer's instructions (including the DNase treatment step). RNA was quantified on a NanoDrop 1000 spectrophotometer (Thermo Fisher Scientific).

### 3.3. Transcriptional profiling

Transcriptional profiling was carried out in a reference style (Cy5-labelled cDNA was derived from RNA, while reference Cy3-labelled cDNA was derived from genomic DNA), as described previously [4]. For each time point, the transcriptional profiles of two biological and two technical replicates were measured. Transition microarray datasets are deposited in ArrayExpress with the accession no. E-MTAB-996. Steady-state transcriptional profiles are available under accession number E-MTAB-285.

### 3.4. RT-PCR

Relative RNA quantities were determined on an Mx3005P Thermocycler using SYBR Green detection of amplification in a two-step protocol. Initially, 2 μg total RNA was converted to cDNA using SuperScriptIII Reverse Transcriptase (Invitrogen) and 1.2 μg Random Primers (Invitrogen) in a 20 μl reaction volume. cDNA was purified using a QiaQuick PCR cleanup column (Qiagen), eluting into 100 μl water. Purified cDNA was analysed (5 μl per well) on a RT-PCR plate using Sensimix dT SYBRGreen Mastermix kit (Quantace) following the manufacturer's instructions (annealing temperature: 58°C; elongation time: 30 s) using primers specific to *ndh, hypB, icd, dmsB* and *hybO*. For normalization, a housekeeping gene *gyrA* was used to control for differences in starting material, while a genomic DNA dilution series was used to correct for differences in primer amplification efficiencies between different primer sets. The sequences of the primer pairs were: *ndh* CGCGACGGGTGTAGAACT, ACGTTCAGGGCTTCGTTG; *gyrA* ACCTTGCGAGAGAAATTACACC, AATGACCGACATCGCATAATC; *hypB* GTCTGGCTGAACGCAACC, AGGCGCATTAGGGTTTCC; *icd* GGCGGTGAACTGATCGAC, GGACGCAGCAGGATCTGT; *dmsB* AGCTTCCGCCGCATTTAT, CGGATCTTCGCAGTGGTT; *hybO* CTGCAGGCGCTATTGGTT, TTCTCCCCGTGAGTCAGC.

### 3.5. Global probabilistic modelling of transcriptomic data

TFInfer [11] was used to deduce the activities of 134 TFs simultaneously. TFInfer is based on a probabilistic model of transcription [8] that relies on a log-linear approximation to transcriptional dynamics. While this is an approximation to the complexity of transcription, it does capture first-order effects and allows inference to be made for a very large number of TFs and genes.

Mathematically, the model is given as follows:
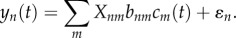
Here *X_nm_* is a binary matrix whose entries are 1 if TF *m* binds gene *n*, and zero otherwise. The 134 TFs in the connectivity matrix were chosen from the EcoCyc database [18] as those with direct evidence of binding to target promoters. The term *b_nm_* accounts for the fact that the effect of a TF is both gene- and transcription-factor-specific; they are given a zero mean Gaussian prior, allowing equal likelihood of repression and activation. The term *c_m_*(*t*) represents the activity of TF *m* at time *t* and is given a state-space model prior that enforces smooth changes in TF activity. Bayesian inference is intractable in this model; so an approximate solution is obtained using a variational mean field approximation.

The model has a clear sign ambiguity as the terms *b_nm_* and *c_m_*(*t*) are multiplied: biologically, it is difficult to distinguish an activator increasing its activity or a repressor decreasing its activity. This ambiguity can be resolved using either prior knowledge of the TF state at some conditions (e.g. FNR being active at the start of an anaerobic to aerobic transition) or by knowing the sign of the interaction of TFs with specific genes.

### 3.6. Differential equation modelling of regulatory circuits

The TFInfer approach is extremely useful to obtain a coarse-grained prediction of regulatory activity across the whole regulatory network. However, it is unlikely to yield accurate predictions of subtler effects such as on/off times of TFs, and it cannot model saturation effects, nonlinearity or mRNA decay. To address these issues, a more detailed model of transcription where the *rate* of mRNA transcription is affected by TFs that exhibit switch-like behaviour was applied. The model was formulated as an ordinary differential equation:
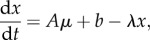
where *x* represents mRNA concentration and **μ** is a binary continuous-time Markov process (telegraph process) representing activation/inactivation of TFs. The time course of the TF activity, along with the kinetic parameters in the right-hand side of the equation, is inferred from the data using a variational Bayesian approximation. The model in this simple single TF form was initially proposed by Sanguinetti *et al*. [19] and later generalized to multiple TFs [10].

### 3.7. NMR

Extracellular metabolite concentrations were measured by ^1^H NMR. Culture supernatants were filtered (0.22 µm pore size) and the resultant cell-free fraction analysed as described previously [4,20].

### 3.8. Purification and phosphorylation of ArcA

ArcA protein was purified as described previously [21]. Phosphorylation was achieved by incubating 4.8 μg His_6_-ArcA in 10 μl of TGED buffer [22] containing 50 mM acetyl phosphate (Sigma) for 30 min at 30°C. The phosphorylated ArcA was used immediately.

### 3.9. *In vitro* transcription assays

These were as described previously [23] except that 1 pmole of σ^70^-saturated *E. coli* RNA polymerase (Epicentre) and 0.1 pmoles of PCR product corresponding to the upstream region of the *ndh* gene were used. This DNA fragment was 426 bp in length and was amplified with the following primers (forward CGAATTCTGTGGGTCGGATA and reverse CAGCTGTGTTGCCATTTCC). Between 0 and 5 μM phosphorylated His_6_-ArcA or 5 μM unphosphorylated His_6_-ArcA was included in each reaction.

### 3.10. Measurement of ArcA phosphorylation

ArcA phosphorylation levels were measured using Phos-tag-acrylamide gel electrophoresis and subsequent Western immunoblotting, as described previously [4].

## 4. Results and discussion

### 4.1. The activities of seven global regulators coordinate gene expression in response to oxygen availability

Transcript profiles were obtained for *E. coli* K-12 cultured in stable environments (i.e. steady states at fixed oxygen supply rates, equivalent to 0, 31, 56, 85, 115 and 217 per cent aerobiosis) [4]. The aerobiosis scale is based on the inverse-linear relationship between oxygen supply and the specific rate of acetate production in glucose-limited chemostat cultures [16]. At 0 per cent aerobiosis, cultures are anaerobic, whereas at 100 per cent aerobiosis, cultures have just sufficient oxygen to grow entirely by aerobic respiration (at aerobiosis values greater than 100%, the oxygen supply exceeds the amount required to support aerobic respiratory growth). The region between 0 and 100 per cent aerobiosis is the micro-aerobic range [16]. The activities of 134 TFs were simultaneously inferred from the global transcript profile of each steady-state using the TFInfer probabilistic modelling software [11]. A brief description of the probabilistic model [8] underlying the TFInfer software is given in §3.5.

**Figure 1.**
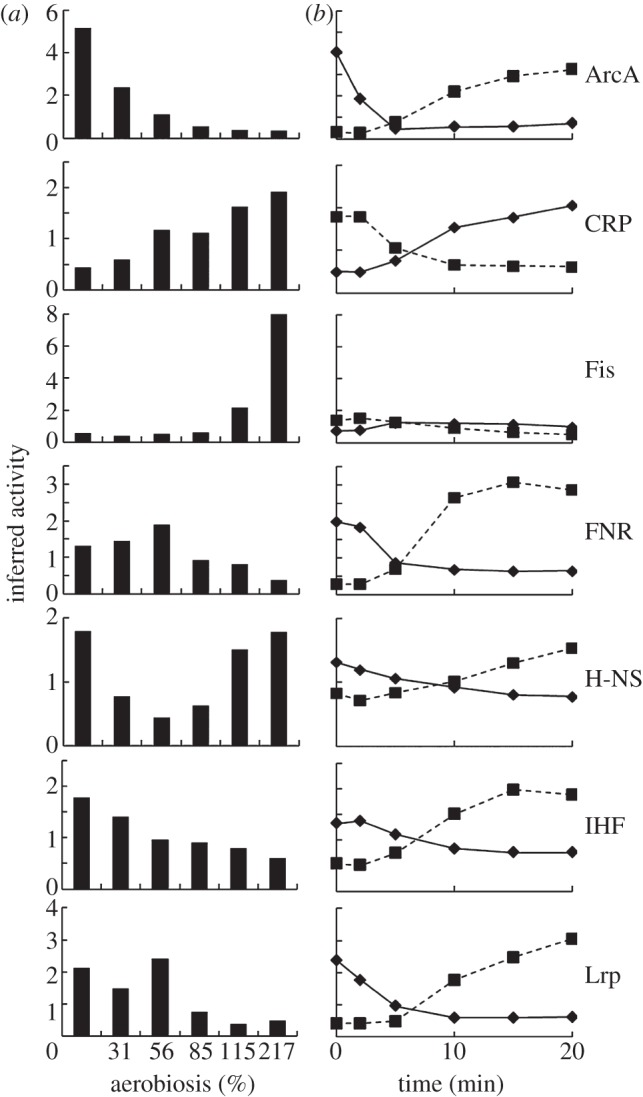
Activity profiles for global TFs predicted from gene expression datasets. (*a*) Inferred activities of the indicated global TFs in stable steady-state cultures grown at defined points on the aerobiosis scale. (*b*) Inferred activities of the global TFs in the unstable environments of transitions from anaerobic to aerobic conditions (diamonds, solid lines) and aerobic to anaerobic conditions (squares, dashed lines). The inferred activities arise from the term *c_m_*(*t*) in the TFInfer model [11]. In all cases, the signal-to-noise ratio was more than 5.

**Figure 2.**
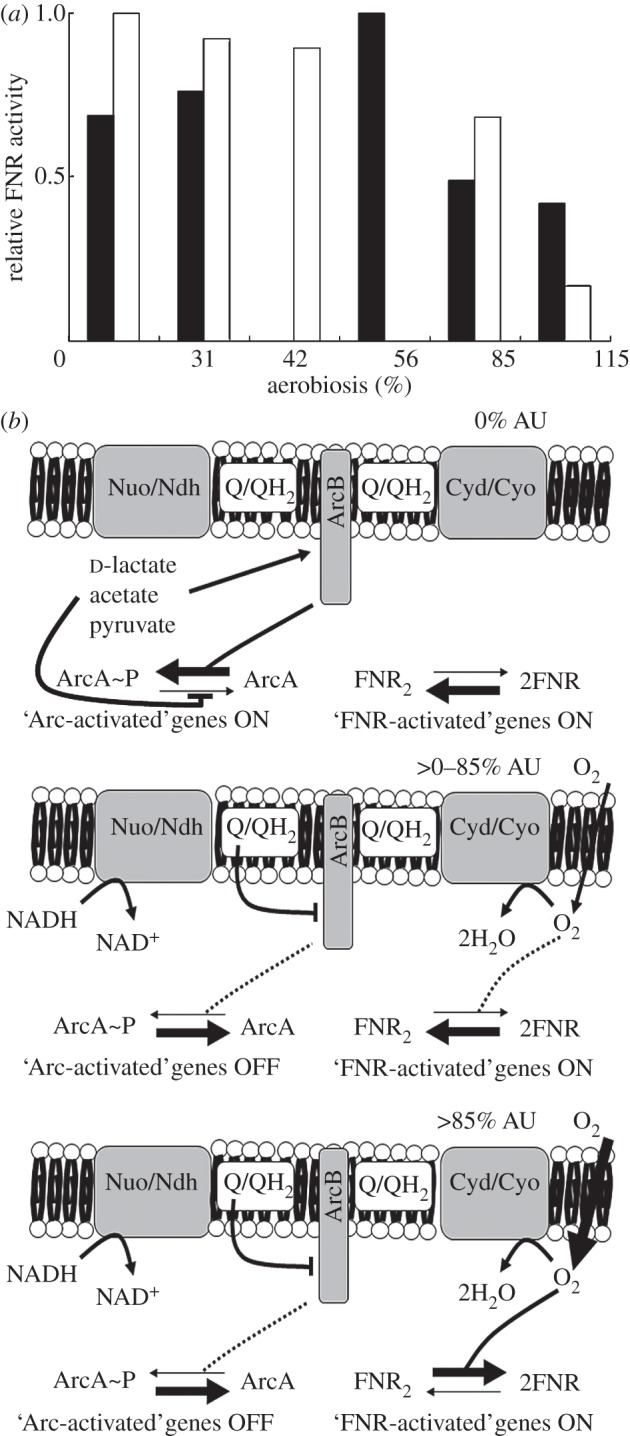
FNR activity under steady-state conditions. (*a*) The relative activity of FNR estimated from measurement of β-galactosidase activities from the model FNR-dependent FF-41.5 promoter fused to *lacZ* (data shown in table 1) in cultures grown at the indicated aerobiosis values (white bars). The black bars show the relative activity of FNR inferred from the transcript profiles at the indicated aerobiosis values (as shown in figure 1). In both cases, 1 represents the maximum activity. In the validation experiments (white bars), the chemostats set to achieve 56 per cent aerobiosis actually reached 42 per cent aerobiosis; thus only a single white bar is shown for 42 per cent aerobiosis and a single black bar for 56 per cent aerobiosis. (*b*) Model illustrating how oxygen consumption at the bacterial cell membrane is sufficient at aerobiosis unit (AU) values less than or equal to 85 per cent to exclude oxygen from the bulk of the cytoplasm. In the absence of oxygen (0% AU), the aerobic electron transport chain is inactive. ArcB autokinase activity is enhanced by: (i) the absence of inhibition by oxidized quinone (Q) [28]; and (ii) fermentation product (d-lactate, acetate, pyruvate) mediated activation of kinase activity and inhibition of ArcA∼P dephosphosphorylation [29,30]. The direct oxygen sensor FNR activates ‘anaerobic’ gene expression in the absence of oxygen [31,32]. Progress up the aerobiosis scale in the greater than 0–85 per cent AU range enhances flux through the aerobic electron transport chain (fed by the major primary dehydrogenases, Nuo and Ndh, and terminating in the major oxidases, Cyd and Cyo) such that oxidized Q is available to inhibit ArcB autokinase activity and the concentrations of fermentation products are lowered, resulting in conversion of ArcA∼P to inactive ArcA. However, FNR remains active, because the abundances of the terminal oxidases [4] are such that oxygen consumption at the membrane protects the cytoplasmic FNR iron–sulphur cluster from oxygen attack (dashed line). Thus, in this ‘micro-aerobic’ range, ArcA-activated genes are switched off but FNR-activated genes remain on. At aerobiosis values greater than or equal to 85 per cent (greater than 85% AU), ArcA is inactivated (dashed line) but the supply of oxygen now exceeds the rate of consumption at the membrane (table 1), exposing FNR to oxygen, thereby switching off FNR-activated genes. Thus, locating sensors in the membrane (ArcB) and the cytoplasm (FNR) allows optimal coordination of gene expression in the ‘micro-aerobic’ range. The model illustrates the data analysis provided in table 1.

In this analysis, an active TF was defined as the DNA-binding form of the protein. There are four recognized mechanisms for modulating TF activity in *E. coli* K-12: ligand binding; covalent modification; sequestration; or altered intracellular concentration. The model implied that the activities of 23 of the 134 TFs were significantly altered (signal-to-noise ratio more than 5) by oxygen availability. Martinez-Antonio & Collado-Vides suggested that global TFs can be identified by the breadth of their regulons, their interactions with co-regulators and alternative σ factors, the number of ‘slave’ TFs, the size of their evolutionary families and the range of conditions under which they are active [24]. By these criteria, ArcA, CRP, Fis, FNR, H-NS, IHF and Lrp were designated as global TFs in *E. coli* K-12 [24]. All seven global TFs exhibited altered activity across the aerobiosis scale (figure 1*a*). The signals perceived by these global TFs are connected to the energetic state of the bacterium; recall that oxygen availability has profound effects on *E. coli* K-12 energetics. Thus, ArcA and FNR regulate respiration in response to oxygen availability (the variable in these experiments), CRP and Lrp sense nutritional state via cAMP and L-leucine concentrations, and Fis, H-NS and IHF modify DNA topology, which is itself dependent on energy status [7,25–27]. The patterns of TF activities indicate a complex interplay between the global regulators to coordinate gene expression across the aerobiosis scale (figure 1*a*). H-NS and Fis were predicted to exhibit more complex activity (DNA-binding) profiles than the other global regulators, with H-NS activity being the lowest in the mid-aerobiosis range and Fis activity being greatly increased under conditions of excess oxygen supply (aerobiosis ≥ 115%). The influence of CRP on the transcriptome progressively increased with increased aerobiosis, whereas the activities of ArcA, FNR, IHF and Lrp decreased as aerobiosis increased. The predicted ArcA activities at set points on the aerobiosis scale have been previously experimentally validated [4]. For FNR, activity was predicted to significantly decrease only at more than or equal to 85 per cent aerobiosis. This behaviour of FNR was experimentally validated by measuring the activity of a synthetic FNR-dependent promoter (figure 2*a* and table 1).

**Table 1.** The capacity for oxygen consumption exceeds oxygen input leading to an anaerobic cytoplasm, as indicated by FNR activity, in micro-aerobic steady-state cultures of *E. coli.*

AU^a^ (%)	(O_2_) in solution^b^ (μM)	O_2_^c^ (× 10^−16^ moles cell^−1^ min^−1^)	Cyd^d^ (× 10^−20^ moles cell^−1^)	Cyo^d^ (× 10^−20^ moles cell^−1^)	maximum potential rate O_2_ consumed by Cyo and Cyd^e^ (× 10^−16^ moles cell^−1^ min^−1^)	FNR-dependent FF-41.5 promoter activity^f^ (Miller units)
0	0	0	1.9	0.72	2.5	5222 ± 171 (100%)
31	0	5.8	8.6	1.0	8.0	4795 ± 170 (92%)
42						4648 ± 149 (89%)
56	0	9.6	11.1	1.3	10.3	
85	0	10.8	11.1	2.3	11.8	3550 ± 232 (68%)
115	15	8.1	3.2	1.9	5.3	864 ± 23 (16%)
217	115	12.6	1.7	1.5	3.6	230 ± 20 (4%)

^a^Aerobiosis units (AU) as defined by Alexeeva *et al*. [16].

^b^Concentration of dissolved oxygen measured by a TruDO dissolved oxygen sensor.

^c^Calculated from the measured number of cells in the steady-state cultures and the oxygen input into the chemostat.

^d^Calculated from the amounts of *bo*′ (Cyo) and *bd* (Cyd) reported by Rolfe *et al*. [4].

^e^Calculated from the Cyo and Cyd values using the data of Kita *et al*. [33,34].

^f^β-Galactosidase activities of steady-state cultures with the FNR-dependent semi-synthetic FF-41.5 promoter fused to *lacZ* to report FNR activity. The values in parentheses are percentage activity assuming that 100% activity is achieved in the absence of oxygen.

### 4.2. Spatial effects can account for the response of FNR in steady-state cultures maintained at fixed points on the aerobiosis scale

The measured concentrations of cytochrome *bo*′ (Cyo—a terminal oxidase with relatively low oxygen affinity, K_M_ ∼ 0.02–0.35 μM) [35] and cytochrome *bd* (Cyd—a terminal oxidase with high affinity for oxygen, K_M_ ∼ 3–8 nM) [36], reported by Rolfe *et al*. [4], suggest that the potential rates of oxygen consumption, calculated from the data of Kita *et al*. [33,34], exceed the rate of oxygen supply until 115 per cent aerobiosis, at which point dissolved oxygen was first detectable in the cultures analysed here (table 1). Thus, it appears that, although oxygen is present at sufficient concentration to alter cell physiology and ArcBA activity at lower aerobiosis values, as evidenced by the altered transcript profiles [4], the bacterial cytoplasm remains essentially anaerobic. The simplest explanation for these observations is that efficient consumption of oxygen by terminal oxidases located at the cell membrane prevents oxygen reaching the bulk of the cytoplasm. This implies that, even on the scale of the bacterial cell, spatial constraints have significant physiological implications. Hence, ‘hybrid’ metabolism, in which anaerobic processes are supported in the cytoplasm while aerobic respiration occurs in the vicinity of the cell membrane, is facilitated under micro-aerobic conditions (figure 2*b*). This concept is consistent with the inferred and measured activity of FNR, the observed production of fermentation products (e.g. acetate), induction of transcripts encoding enzymes of the glyoxylate shunt in the microarray data (implying that at least some excreted acetate is used) and the absence of detectable dissolved oxygen under micro-aerobic conditions (table 1) [4]. Furthermore, the spatial separation of oxygen consumption and FNR sensing of oxygen could provide an explanation for the presence of membrane-associated (ArcBA) and cytoplasmic (FNR) TFs to coordinate global gene expression in response to oxygen availability. Hence, the membrane-bound sensor of respiratory activity and anaerobic metabolism ArcB [28–30] mediates changes in gene expression through its cognate regulator ArcA when the supply of oxygen is insufficient to fully penetrate the cytoplasm and inactivate the direct oxygen sensor FNR [31,32]. Further work is now needed to test this hypothesis and establish the relative contributions of the alternative terminal oxidases in shielding transcriptionally active FNR from inactivation.

### 4.3. Perturbation of steady-state cultures identifies a core oxygen-responsive TF network

As a commensal enteric bacterium *E. coli* encounters a wide range of environments during transit through a host digestive system to the outside world. A central feature of this lifestyle is the transition between anaerobic (e.g. host intestine) and aerobic (e.g. external milieu) environments. To determine the response of the seven global TFs in environments in which the oxygen supply is either increasing or decreasing, steady-state chemostat cultures were sampled for transcript profiling before perturbation by altering the gas mix used to sparge the cultures. After 2, 5, 10, 15 and 20 min, further samples were obtained for transcript profiling. For the anaerobic–aerobic transition, the dissolved oxygen tension was initially zero and rose after 1 min to 40 per cent saturation, and after 2 min it stabilized at 95 per cent saturation. In the aerobic–anaerobic transition, the dissolved oxygen tension was initially 65 per cent and fell after 1 min to 29 per cent, and after 2 min stabilized at 0 per cent. Thus, the rate of change in oxygen availability for the two transitions was similar, but with opposite sign.

All seven global TFs discussed already responded in both transitions. In all cases, the responses were reversible during the acute phase of adaptation in that where activity was predicted to increase in the aerobic–anaerobic transition (figure 1*b*, squares, dashed lines), it was predicted to decrease in the anaerobic–aerobic transition, and vice versa (figure 1*b*, diamonds, solid lines).

**Figure 3.**
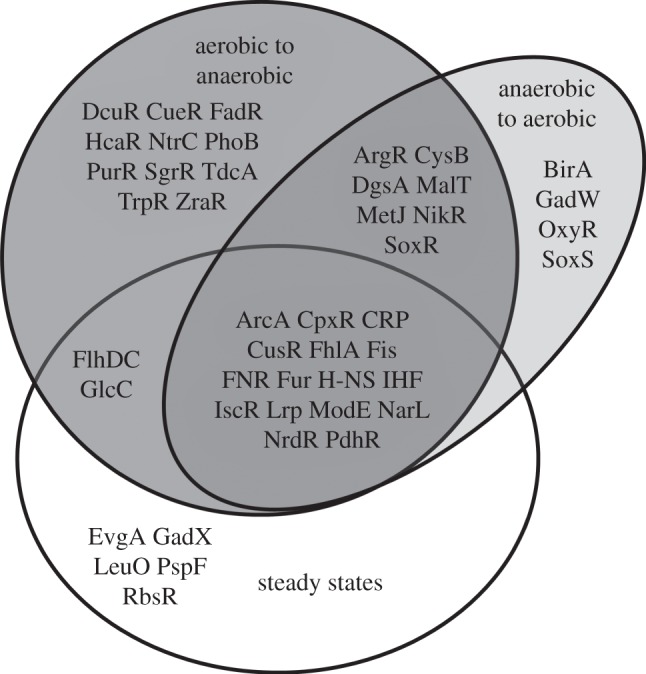
The oxygen-responsive TF network of *E. coli* K-12. The three ovals represent the steady-state cultures, the anaerobic–aerobic transition and the aerobic–anaerobic transition. TFs that are predicted to respond are indicated. Sectors that overlap contain TFs that respond in two or all three of the conditions tested.

The inferred responses of the global TFs are consistent with them acting as part of a core network in coordinating gene expression in response to changes in oxygen availability (figure 3). This core network was extended to include local regulators (CpxR, CusR, FhlA, Fur, IscR, ModE, NarL, NrdR and PdhR) that exhibited responses in the stable environments of steady-state cultures across the aerobiosis scale and in the unstable environments of the transitions (figure 3). Thus, changes in oxygen availability (sensed directly by FNR, and indirectly by ArcA) affect the redox state of the system, leading to consequences for metal ion homeostasis (copper, molybdate, iron; sensed by CusR, ModE and Fur), iron–sulphur cluster turnover (sensed by IscR), ribonucleotide reductase activity (regulated by NrdR), over-metabolite production (formate and pyruvate; sensed by FhlA and PdhR) and cell-envelope stress (sensed by CpxR). Therefore, it is clear that the influence of oxygen availability on the *E. coli* K-12 transcriptome extends beyond those genes controlled by the known oxygen-responsive regulators, ArcA and FNR, and it is suggested that the activity of a core network of 16 TFs is modulated to coordinate *E. coli* K-12 gene expression in environments with different, but stable, oxygen availabilities or in the unstable environments of the transitions (figure 3).

A comparison of the TF responses for the steady-state and transient cultures revealed that changes in activities were apparent in the latter but not in the former, and vice versa. Thus, additional TFs interact with the core network to integrate the response to specific signals into the transcriptional programming of the bacterium (figure 3). For example, in steady-state cultures, as aerobiosis increased, the excretion of acidic fermentation end-products decreased [4], and the acid responsive regulators EvgA and GadX exhibited correspondingly lower activities (figure 3). These TFs did not feature in either transition because there was insufficient time for the acidic end-products to sufficiently accumulate (aerobic to anaerobic) or diminish (anaerobic to aerobic) to affect their activity (table 2). OxyR and SoxS are the major regulators of the oxidative stress response in *E. coli* K-12 [37]. Both these TFs were predicted to respond only during the anaerobic to aerobic transition (figure 3), presumably reflecting the need to manage a burst of reactive oxygen species (peroxide and superoxide) specifically during adaptation to aerobic growth.

**Table 2.** Measurements of extracellular metabolites during transitions of *E. coli* MG1655 between environments with different oxygen availabilities.

time after transition (min)	acetate (mM)	formate (mM)	succinate (mM)	pyruvate (mM)	lactate (mM)	fumarate (mM)	ethanol (mM)
to aerobic conditions							
0	17.9 ± 0.2	35.3 ± 0.3	4.6 ± 0.1	<0.10	0.19 ± 0.04	0.03 ± 0.01	9.5 ± 0.02
20	19.0 ± 0.2	36.2 ± 0.6	4.3 ± 0.1	1.1 ± 0.01	0.29 ± 0.01	0.17 ± 0	9.8 ± 0.2
to anaerobic conditions							
0	<0.10	<0.1	<0.05	<0.10	<0.05	<0.01	<0.10
20	1.37 ± 0.1	2.6 ± 0.1	0.36 ± 0.6	<0.10	<0.05	<0.01	0.98 ± 0.07

Thus, it is concluded that ArcA, CRP, Fis, FNR, H-NS, IHF and Lrp are truly global regulators, at least as far as the response to oxygen availability is concerned, in the sense that their outputs create the transcriptional framework upon which local regulators operate in response to specific unstable environments.

### 4.4. PdhR behaviour during transitions accounts for the irreversibility of the *ndh* transcript profile

**Figure 4.**
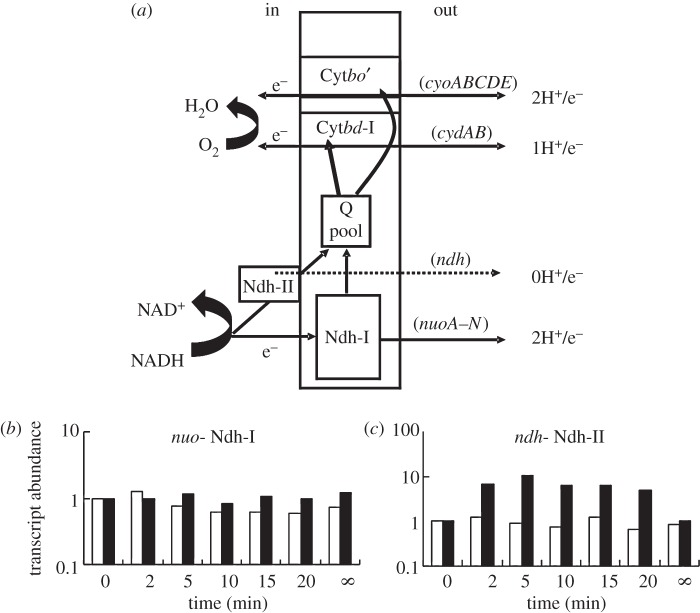
Profiles of transcripts encoding alternative NADH dehydrogenases during adaptation to changes in oxygen availability. (*a*) Diagram showing the components of the best characterized branched aerobic electron transport chain of *E. coli*. The central rectangle represents the cytoplasmic membrane. NADH is oxidized by either the proton-translocating NADH dehydrogenase I (Ndh-I, solid line) or by the non-proton-translocating NADH dehydrogenase II (Ndh-II, broken line). Electrons are fed into the quinone pool (Q) and then used by the terminal oxidases (cytochrome *bd*, Cyt *bd*-I; or cytochrome *bo′*, Cyt *bo′*) in the reduction of oxygen to water. Cyt *bo′* has a relatively low affinity for oxygen, whereas Cyt *bd*-I has a higher affinity for oxygen. Because of the different properties of the dehydrogenases and the oxidases, between one and four protons can be translocated for each electron. (*b*,*c*) Transcript profiles of (*b*) *nuoA-N* and (*c*) *ndh*, during aerobic–anaerobic (white bars) and anaerobic–aerobic (black bars) transitions. Time zero is the aerobic steady state (white bars) or the anaerobic steady state (black bars). The infinity symbol represents the final steady state (anaerobic for the white bars and aerobic for the black bars).

One of the key adaptations made by *E. coli* in response to altered oxygen availability is switching between alternative aerobic electron transport chains by ‘mixing and matching’ combinations of primary dehydrogenases (e.g. NADH dehydrogenase I, Ndh-I, encoded by the *nuo* operon, and NADH dehydrogenase II, Ndh-II, encoded by *ndh*) and terminal oxidases (e.g. cytochrome *bo′*, encoded by the *cyoA-E* operon, and cytochrome *bd*-I, encoded by the *cydAB* operon) to optimize energy conservation and growth potential (figure 4*a*).

**Figure 5.**
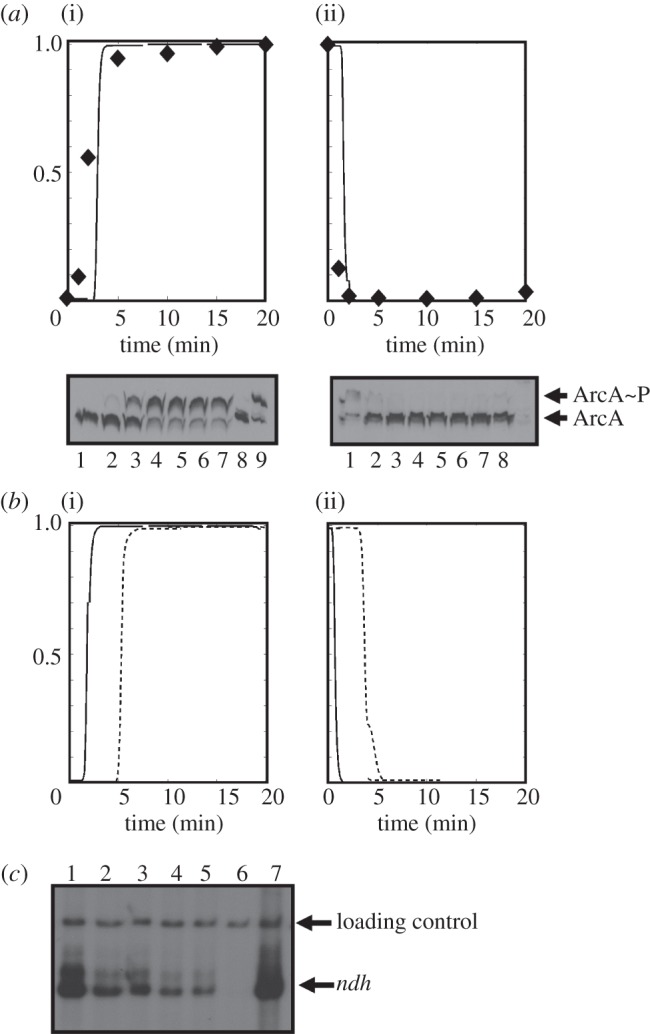
ArcA and FNR activities during transitions between aerobic and anaerobic conditions, and repression of *ndh* transcription by ArcA *in vitro.* (*a*) Predicted activities of ArcA (solid lines) during transition from aerobic to anaerobic (i) and anaerobic to aerobic (ii) conditions. The ordinate axes are the ArcA activities (0–1, where 0 is off and 1 is on) estimated from a model based on a two-state Markov jump process in which the TF activity moves quickly between on and off states. To validate the model, the phosphorylation state of ArcA in the bacterial cells at the indicated times was determined by quantitative densitometry of Western blots (representatives shown below the charts) of ArcA separated by Phos-tag-acrylamide gel electrophoresis [4] 0, 1, 2, 5, 10, 15 and 20 min into the transitions (lanes 1–7). Lane 8 shows purified unphosphorylated His-tagged ArcA, and lane 9 shows the phosphorylated form (ArcA∼P) [4]. For each transition, the maximum amount of ArcA∼P measured was set at 1 so that relative ArcA activity could be calculated (diamond data points on the charts) for comparison with the model. (*b*) Predicted activities of FNR (solid lines) during transition from aerobic to anaerobic (i) and anaerobic to aerobic (ii) conditions. The ordinate axes are the FNR activities (0–1, where 0 is off and 1 is on). To validate the model, the activity of FNR was estimated by measuring transcription from a single-copy synthetic FNR-dependent promoter by RT-PCR (dashed lines). (*c*) *In vitro* transcription of *ndh* in the absence and presence of ArcA. Lanes 1–6, 0, 0.8, 1.3, 1.9, 2.5, 5.0 µM ArcA∼P; lane 7, 5.0 µM dephosphorylated ArcA. The locations of the *ndh* transcript and the loading control are indicated.

In both transitions, the responses of the *nuo* operon transcripts encoding the energy conserving Ndh-I were minimal (figure 4*b*). By contrast, the *ndh* transcript, encoding the non-proton translocating Ndh-II, responded strongly in the transition from anaerobic to aerobic conditions (figure 4*c*, black bars), but not in the reverse transition (figure 4*c*, white bars). Transcription of *ndh* is repressed by FNR [38] and gene expression profiling suggests that it is activated by ArcA [39]. Therefore, to determine whether the observed asymmetry of the *ndh* response could be accounted for by changes in the activities of ArcA and FNR, a dynamical model was developed based on high-resolution RT-PCR data for a training set of genes that were either ArcA- (*hypB* and *icd*) or FNR- (*dmsB*, *hybO*) regulated. Details of the modelling procedure are given in §3.6. This model is based on a differential equation representation of the system [10], yielding more realistic estimates for the response times of ArcA and FNR in the transitions than could be obtained from the low-resolution global transcript profiling data. The model implied that in both transitions the response times of ArcA (figure 5*a*, solid lines) and FNR (figure 5*b*, solid lines) were similar. The modelled behaviours of ArcA and FNR were validated by direct measurements of the abundance of phosphorylated ArcA (figure 5*a*, diamonds) and by quantifying the transcript generated from activation of the synthetic FNR-dependent promoter FF-41.5 (figure 5*b*, dashed lines) during the transitions. The predicted profiles show a remarkable symmetry in that response times of both TFs are similar in both directions. Therefore, a model based solely on ArcA and FNR control could not account for the observed asymmetry in the *ndh* expression profile. Moreover, *in vitro* transcription reactions showed that rather than activating *ndh* expression phosphorylated ArcA acted as a repressor (figure 5*c*). This latter observation is consistent with the locations of the predicted ArcA binding sites in the *ndh* gene (one upstream of the transcript start at −57; and five downstream at +36, +52, +57, +66 and +68) [18]. Therefore, the dynamical behaviour and regulatory outputs of ArcA and FNR cannot account for the asymmetric nature of the *ndh* transcript profile in the transitions.

**Figure 6.**
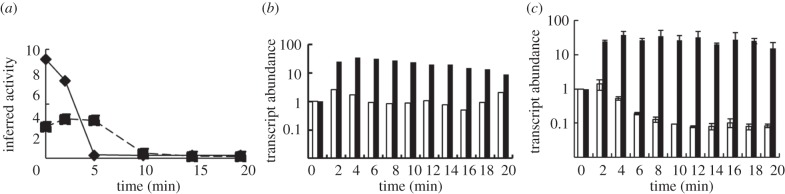
The PdhR response accounts for the asymmetrical behaviour of the *ndh* transcript in transitions. (*a*) The inferred activity of PdhR during anaerobic–aerobic (diamonds, solid line) and aerobic–anaerobic (squares, dashed line) transitions. High-resolution RT-PCR data for the *ndh* transcript: (*b*) wild-type *E. coli* K-12; (*c*) *pdhR* mutant, during aerobic–anaerobic (white bars) and anaerobic–aerobic (black bars) transitions.

In addition to regulation by ArcA and FNR, *ndh* expression is repressed by the pyruvate-responsive TF PdhR; repression is relieved by pyruvate [40]. The TFInfer model implied that the PdhR response was fast and strong upon transfer of anaerobic cultures to aerobic conditions, consistent with the presence of pyruvate in the culture medium from this transition (figure 6*a*, diamonds, solid line; table 2). In the reverse transition, the PdhR response was relatively slow and weaker, consistent with the failure to detect pyruvate excretion (table 2), presumably because when PFL is synthesized, pyruvate is rapidly consumed. But, crucially, the PdhR response was predicted to have the same sign in both transitions (figure 6*a*)—that is, in both anaerobic–aerobic (figure 6*a*, diamonds, solid line) and aerobic–anaerobic (figure 6*a*, squares, dashed line)—and PdhR activity was predicted to decrease. Thus, the model generated the hypothesis that PdhR was the source of the asymmetrical *ndh* transcript profiles in the transitions. To test this, high-resolution (every 2 min for 20 min) RT-PCR measurements of *ndh* transcript abundance in wild-type *E. coli* cultures were obtained. These measurements (figure 6*b*) were qualitatively similar to those obtained by microarray analysis (figure 4*c*). Thus, *ndh* expression was rapidly and strongly enhanced in the anaerobic to aerobic transition, which could be explained by ArcA, FNR and PdhR all repressing *ndh* expression under the initial anaerobic conditions followed by rapid de-repression upon introduction of oxygen, which inactivates ArcA and FNR, and causes pyruvate accumulation, by inhibition of pyruvate formate-lyase [20], to inactivate PdhR. Despite the activation of the repressors ArcA and FNR by the withdrawal of oxygen in the aerobic–anaerobic transition, *ndh* expression did not decrease (figure 6*b*), consistent with the microarray data (figure 4*c*). The predicted activity of PdhR (figure 6*a*) implied that it slowly switched to a lower activity during the transition. This would de-repress *ndh* expression to oppose ArcA and FNR-mediated repression, maintaining relatively constant *ndh* expression. This explanation of the asymmetry of the *ndh* transcript profiles in the transitions implies that the *ndh* transcript response in a *pdhR* mutant should resemble the wild-type for the anaerobic–aerobic transition (*ndh* repression is still relieved upon inactivation of ArcA and FNR). By contrast, the *ndh* transcript abundance should decrease in aerobic–anaerobic transitions of the *pdhR* mutant because there is no PdhR-mediated de-repression to counter-balance ArcA and FNR-mediated repression that occurs upon oxygen exposure. Therefore, in the *pdhR* mutant the *ndh* transcript profile was predicted to be reversible. This proved to be the case (compare figure 6*b,c*). Thus, changes in PdhR activity account for the asymmetric behaviour of the *ndh* transcript during transitions between environments with different oxygen availabilities. This observation emphasizes the need to examine dynamic as well as steady-state behaviour to fully understand adaptive processes mediated by gene regulatory circuits in bacteria.

### 4.5. Conclusion

A core network of TFs that includes the major oxygen-responsive regulators ArcA and FNR has been identified that controls transcriptional adaptation when *E. coli* encounters environments with different oxygen availabilities (figures 1 and 3). The predicted ArcA [4] and FNR activities have been experimentally verified (figure 2*a*) and are indicative of significant spatial effects that affect the reprogramming of gene expression in the micro-aerobic range. Thus, cytoplasmic FNR (and other oxygen-sensitive proteins) are protected by oxygen consumption at the membrane by the terminal oxidases—a form of respiratory protection that allows ‘hybrid’ anaerobic and aerobic metabolism to function under micro-aerobic conditions (figure 2*b* and table 1). The core oxygen-responsive TF network is extended by additional TFs that operate to manage specific challenges posed by particular steady-state or dynamic conditions. The mechanistic insight that can be gained by applying the systems approach advocated here is clearly illustrated by identifying the TF PdhR as the source of the asymmetric behaviour of the *ndh* transcript in transitions (figures 4 and 6). Thus, the combination of transcript profiling in stable and dynamic environments, mathematical modelling, and biochemical measurements demonstrates the added value of a systems approach to fully appreciate and understand the mechanisms underpinning fundamental adaptive processes in bacteria.
